# Tissue discrimination by bioelectrical impedance during PLL resection in anterior decompression surgery for treatment of cervical spondylotic myelopathy

**DOI:** 10.1186/s13018-019-1380-x

**Published:** 2019-11-06

**Authors:** Fuqiang Shao, He Bai, Muyao Tang, Yuan Xue, Yu Dai, Jianxun Zhang

**Affiliations:** 1 Department of Orthopedics Surgery, Tianjin Medical University General Hospital, 154 Anshan Road, Heping District, 300052 Tianjin, People’s Republic of China; 2 Department of Orthopedics Surgery, Inner Mongolia Cancer Hospital & Affiliated People’s Hospital of Inner Mongolia Medical University, 42 Zhao wuda Road, Hohhot, 010020 Inner Mongolia People’s Republic of China; 3 Experimental Trauma Surgery, Justus-Liebig University Giessen, Aulweg 128, 35392 Giessen, Germany; 4 Institute of Robotics and Automatic Information System, Tianjin Key Laboratory of Intelligent Robotics, College of Computer and Control Engineering, Nankai University, 94 Weijin Road, Nankai District, 300071 Tianjin, People’s Republic of China; 5 Tianjin Key Laboratory of Spine and Spinal Cord, Tianjin Medical University General Hospital, Tianjin, People’s Republic of China

**Keywords:** Bioelectrical impedance, Tissue discrimination, Posterior longitudinal ligament, Anterior cervical discectomy and fusion, Cervical spondylotic myelopathy, Robot-assisted minimally invasive surgery

## Abstract

**Background:**

The electrical properties of biological tissues differ depending on their physical properties. This study aimed to explore if bioelectrical impedance (modulus and phase) would discriminate tissues relevant to resection of the posterior longitudinal ligament (PLL) in anterior cervical decompression surgery.

**Methods:**

PLL resection via an anterior approach was performed on the C4/5 segments in six mini-pigs. The bioelectrical impedance measurements were performed for two tissue groups (annulus fibrosus, endplate cartilage, sub-endplate cortical bone, and PLL; PLL, dura mater, spinal cord, and nerve root) using a novel probe and a precision inductance-capacitance-resistance meter. For each group, impedance was analyzed in terms of modulus and phase along a broad spectrum of frequencies (200–3000 kHz) using a nonparametric statistical analysis (Kruskal-Wallis).

**Results:**

The analysis showed a clear difference among the tissues. The modulus and phase show the same changing trend with frequency and present lower values at higher frequencies. Among annulus fibrosus, endplate cartilage, sub-endplate cortical bone, and PLL, it was possible to discriminate each tissue at every frequency point, considering the phase (*p* < 0.05), while this was not always the case (i.e., annulus fibrosus vs PLL at frequency of 200 kHz, 400 kHz, and 3000 kHz, *p* > 0.05) for modulus. Among PLL, dura mater, spinal cord, and nerve root, for every comparison, a statistically significant difference was reported in the modulus, phase, or both (*p* < 0.05).

**Conclusions:**

The results indicated the potential of bioelectrical impedance to provide real-time tissue differentiation and enhance safe PLL resection in anterior cervical decompression surgery, particularly in robot-assisted minimally invasive surgery (RMIS).

## Background

Cervical spondylotic myelopathy (CSM) is the commonest type of spinal cord dysfunction among patients older than 55 years and the commonest cause of acquired spastic paraparesis in the middle and later years of life, causing threat to human health [1–4]. Anterior cervical decompression is one of the most common surgical procedures adopted in the treatment of CSM [5, 6].

Disc removal followed by posterior longitudinal ligament (PLL) resection has been advocated for removing the hypertrophic PLL and achieving an increase in diameter of the spinal cord during anterior decompression in the cervical spine [7–9]. However, PLL resection is a technical challenge owing to the potential risks of complications such as dual defects, cerebrospinal fluid leakage, and injury to the spinal cord or nerve root [10–13]. It is therefore important to establish a suitable method for discriminating the PLL and dura during the conventional decompression surgery. Tissue discrimination by impedance would provide real-time values and offer a simple auxiliary feedback system for PLL resection in anterior cervical decompression surgery, particularly in robot-assisted minimally invasive anterior cervical discectomy and fusion (ACDF) surgery.

Bioelectrical impedance analysis is a technique that exploits the electrical properties of biological organs and tissues to indicate their physical properties. Bioelectrical impedance measurement is non-invasive, simple, and shows adequate repeatability at a relatively low cost [14]. Tissue discrimination based on bioelectrical impedance has been frequently reported in different clinical settings [14–16]. However, there are few reports on tissue discrimination using bioelectrical impedance during resection of PLL in ACDF surgery for CSM.

In this study, we measured bioelectrical impedance of different tissues in vivo relevant to PLL resection during ACDF surgery and offered a potentially auxiliary tissue discrimination system for use in robot-assisted minimally invasive ACDF surgery.

## Materials and methods

### Animals

Experiments were performed at the Department of Anatomy of Tianjin Medical University and in accordance with the guidelines for animal care. All animal experimental procedures were approved by the Animal Ethics Committee of Tianjin Medical University.

Six mini-pigs that were obtained from the Experimental Animal Center of Tianjin Medical University underwent PLL resection via the anterior approach in the cervical spine. The general characteristics of the animals are shown in Table 1. Animals were acclimatized at the Animal Research Facility for a period of 48 h before experimentation and fed a regular diet ad libitum.

**Table 1:** The general characteristics of the animals

Total number of mini-pigs	6
Sex	
Male	3
Female	3
Age (month, range, mean ± SD)	8.0–9.0, 8.4 ± 0.34
Weight (kg, range, mean ± SD)	27–33, 29.7 ± 2.3

*SD* standard deviation

### Surgical procedures

**Fig. 1:**
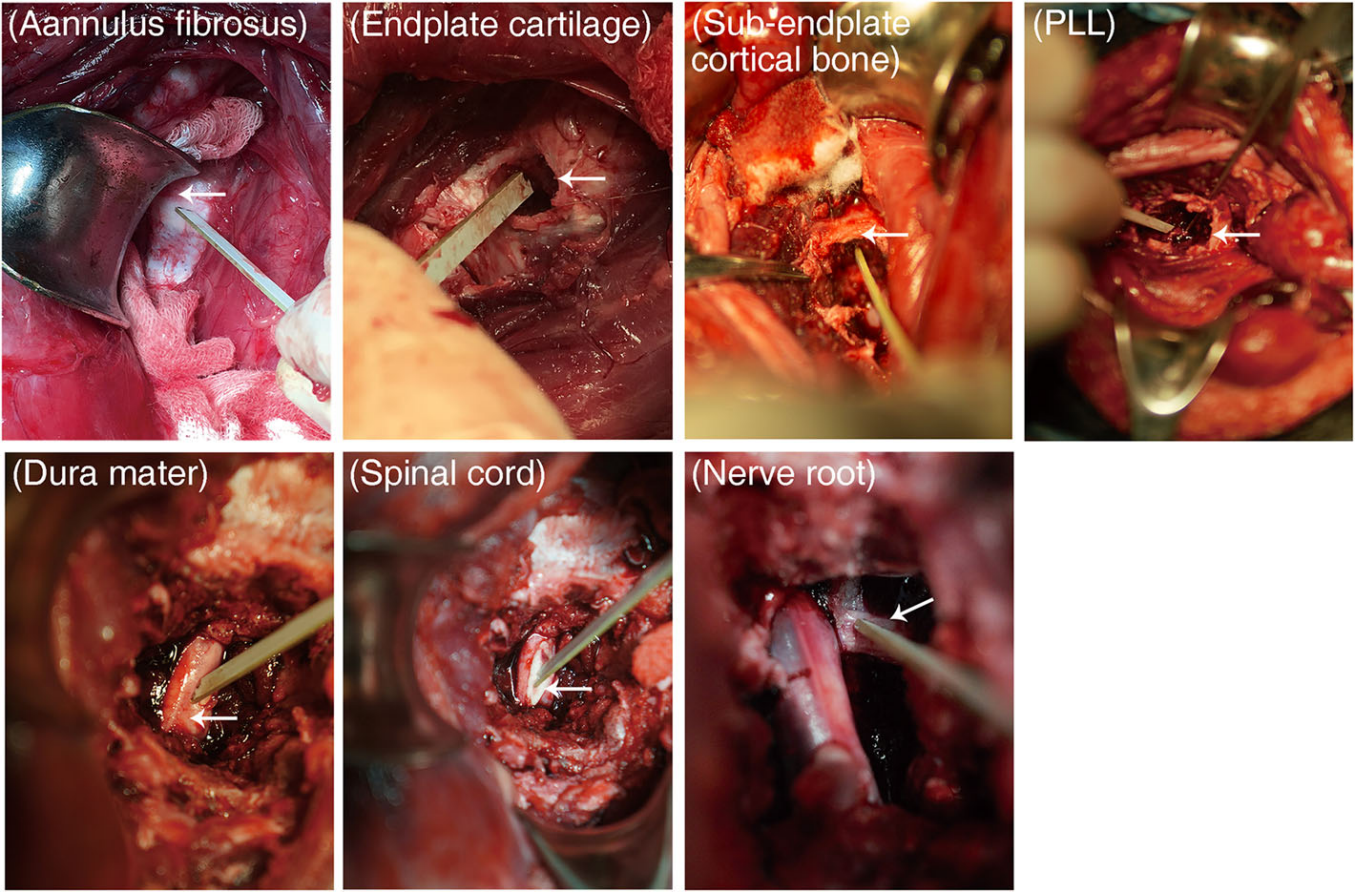
PLL resection via anterior approach on the C4/5 segment and bioelectrical impedance measurement of annulus fibrosus, endplate cartilage, sub-endplate cortical bone, posterior longitudinal ligament, dura mater, spinal cord, and nerve root

Under general anesthesia by intravenous infusion of 3% sodium pentobarbital (30 mg/kg), the animals were fixed on an animal operating table in the supine position with the neck slightly extended. A midline longitudinal skin incision was made in the submandibular region. The incision was extended vertically to provide adequate exposure if necessary. After necessary discectomies, the C4/5 intervertebral disc and endplate cartilage were removed using appropriate curette or vessel forceps until the PLL and sub-endplate cortical bone was exposed. Thereafter, the PLL was resected to expose the dura mater. Part of the dura mater was removed for measurement of the bioelectrical impedance of the spinal cord and nerve root (Fig. 1).

### Biolectrical impedance measurement

**Fig. 2:**
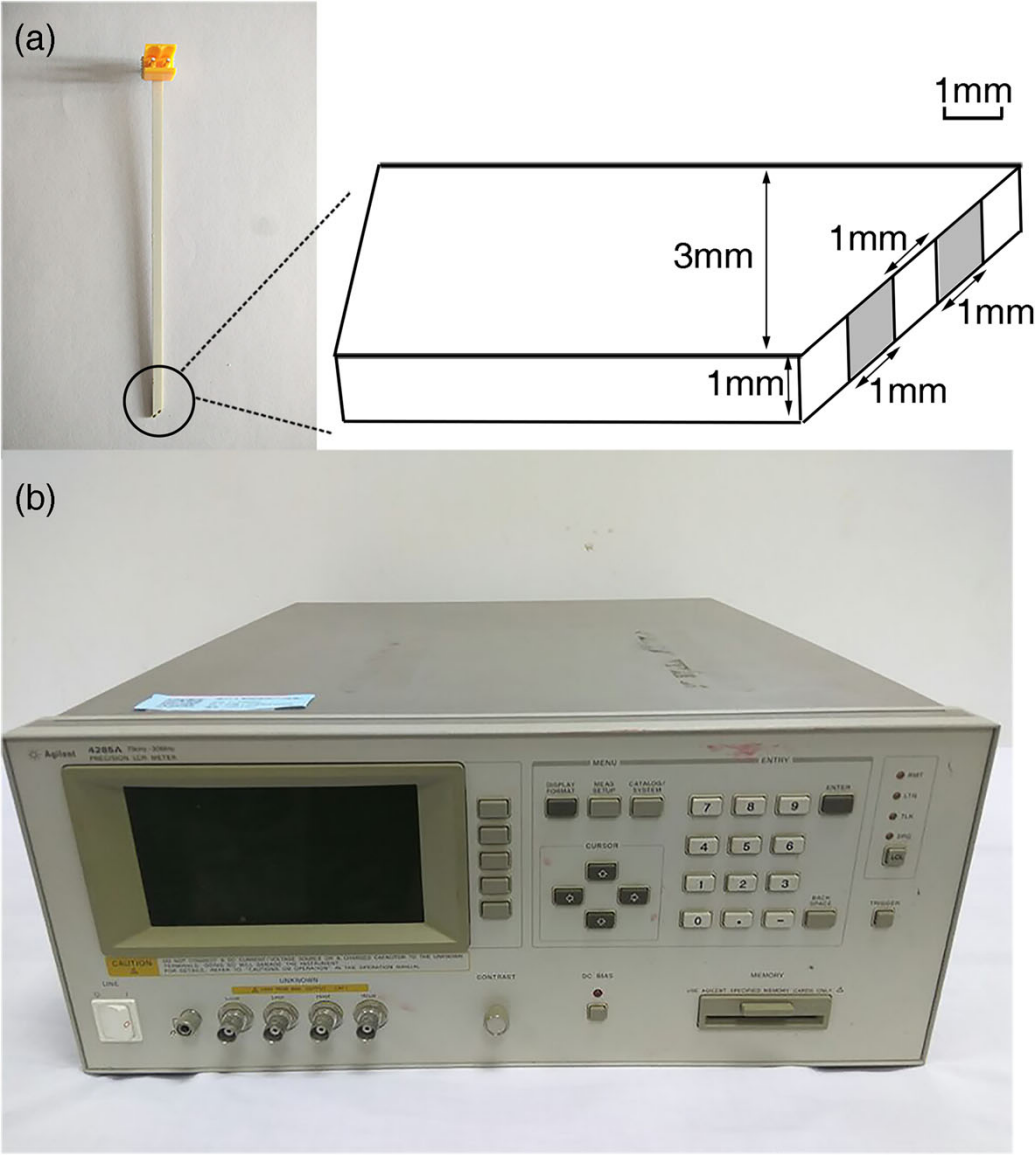
The bioelectrical impedance measurements apparatus. **a** The structure drawing of the custom probe. **b** The inductance-capacitance-resistance meter

The bioelectrical impedance measurement apparatus consisted of a custom probe and an inductance-capacitance-resistance (LCR) meter (4285A; Agilent, Santa Clara, CA). The probe (Fig. 2a) was 100 mm long, 3 mm wide, 1 mm thick, and completely covered with insulating material except for the tip. The recording sites were two electrodes, each with an area of 1 mm × 1 mm and a distance of 1 mm between the electrodes. By applying a known current (0.1 mA) between the two electrodes, the voltage between the electrodes was measured and the electrical impedance was calculated. The LCR meter (Fig. 2b) with a sampling frequency of 1 Hz and a general purpose interface bus interface were applied to measure the complex impedance (modulus and phase) of the tissue under examination. Because of polarization impedance, a frequency range of 200 kHz to 3000 kHz was selected and the frequency points were 200 kHz, 400 kHz, 600 kHz, 800 kHz, 1000 kHz, 2000 kHz, and 3000 kHz.

The frequency range belongs to the beta dispersion region, which contains information about both the extra- and intracellular environments, making it well suited for discriminating different tissues.

The bioelectrical impedance of all relevant tissues including annulus fibrosus, endplate cartilage, sub-endplate cortical bone, PLL, dura mater, spinal cord, and nerve root was measured. Impedance reproducibility using the same probe was assessed for each tissue with five measurements in two different locations, thus obtaining 10 measurements (expressed in terms of modulus and phase). After each measurement, the probe was washed with saline solution (0.9% sodium chloride) and wiped with a piece of gauze. During the measurements, the probe was kept in contact with the tissue surface by a constant force and removed after completing each measurement. The bioelectrical impedance measurements were also repeated by three experienced surgeons at a room temperature of 25 °C to minimize the measurement error.

### Analysis

The bioelectrical impedance values were downloaded into a Microsoft Excel spreadsheet and transferred to IBM SPSS Statistics version 22 (SPSS, Inc., Chicago, IL, USA) for statistical analyses. Data were shown as mean ± standard deviation (SD). As impedance data were not normally distributed, nonparametric statistical analyses were performed on modulus and phase to investigate the significance of the differences among the tissues. The comparison was performed along the entire frequency spectrum on the in vivo data by dividing it into two groups: annulus fibrosus, endplate cartilage, sub-endplate cortical bone, and PLL; PLL, dura mater, spinal cord, and nerve root. Statistical Kruskal-Wallis one-way analysis of variance was performed as post hoc tests. The level of significance was set at *p*< 0.05 for all statistical analyses.

## Results

**Fig. 3:**
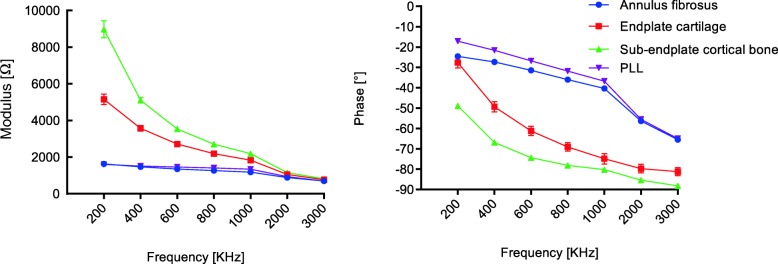
Bioelectrical impedance at different frequencies of modulus and phase: mean ± standard deviation data of annulus fibrosus, endplate cartilage, sub-endplate cortical bone, and posterior longitudinal ligament

**Fig. 4:**
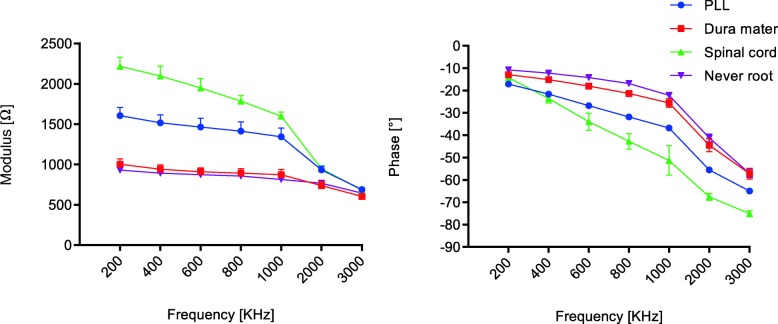
Bioelectrical impedance at different frequencies of modulus and phase: mean ± standard deviation data of posterior longitudinal ligament, dura mater, spinal cord, and nerve root

The global behavior of the measurements on the six mini-pigs was managed and analyzed. The following graphs (Figs. 3 and 4) show the mean value of each tissue group, considering the 60 sets of measurement values, with the respective SD of modulus and phase along the whole frequency spectrum. Tables 2 and 3 show this in more detail.

**Table 2:** Modulus and phase of tissues in the first group at different frequencies (mean ± SD)

Tissues	Frequency (kHz)						
	200	400	600	800	1000	2000	3000
AF, modulus, Ω	1633.14 ± 125.19	1469.3 ± 120.42	1348.54 ± 111.07	1265.77 ± 83.75	1184.74 ± 71.02	881.82 ± 28.61	703.24 ± 12.42
AF, phase, °	− 24.55 ± 0.44	− 27.34 ± 0.42	− 31.47 ± 0.43	− 35.99 ± 0.44	− 40.37 ± 0.45	− 56.34 ± 0.4	− 65.51 ± 0.47
EC, modulus, Ω	5157.27 ± 278.7	3573.47 ± 152.34	2712.78 ± 92.09	2190.76 ± 73.36	1835.17 ± 60.89	1053.72 ± 19.08	769.21 ± 9.54
EC, phase, °	− 27.65 ± 2.56	− 49.34 ± 2.48	− 61.2 ± 2.25	− 69.04 ± 2.01	− 74.9 ± 2.56	− 79.72 ± 2.07	− 81.18 ± 1.97
SC, modulus, Ω	8974.01 ± 459.7	5108.27 ± 147.23	3538.45 ± 95.82	2705.01 ± 69.12	2193.67 ± 51.13	1156.27 ± 23.65	814.01 ± 16.15
SC, phase, °	− 48.87 ± 0.43	− 66.84 ± 0.45	− 74.33 ± 0.46	− 78.12 ± 0.45	− 80.23 ± 0.5	− 85.4 ± 0.46	− 88.23 ± 0.46
PLL, modulus, Ω	1605.88 ± 100.73	1515.69 ± 101.56	1463.82 ± 108.16	1412.2 ± 118.24	1344.05 ± 106.08	932.2 ± 44.16	688.25 ± 28.12
PLL, phase, °	− 17.1 ± 0.46	− 21.6 ± 0.44	− 26.81 ± 0.42	− 31.83 ± 0.43	− 36.75 ± 0.45	− 55.53 ± 0.47	− 64.95 ± 0.42

*SD* standard deviation, *AF* annulus fibrosus, *EC* endplate cartilage, *SC* sub-endplate cortical bone, *PLL* posterior longitudinal ligament

**Table 3:** Modulus and phase of tissues in second group at different frequencies (mean ± SD)

Tissues	Frequency (kHz)						
	200	400	600	800	1000	2000	3000
PLL, modulus, Ω	1605.88 ± 100.73	1515.69 ± 101.56	1463.82 ± 108.16	1412.2 ± 118.24	1344.05 ± 106.08	932.2 ± 44.16	688.25 ± 28.12
PLL, phase, °	− 17.1 ± 0.46	− 21.6 ± 0.44	− 26.81 ± 0.42	− 31.83 ± 0.43	− 36.75 ± 0.45	− 55.53 ± 0.47	− 64.95 ± 0.42
DM, modulus, Ω	1002.52 ± 67.46	941.9 ± 52	909.5 ± 47.42	892.12 ± 55.52	873.33 ± 65.33	738.49 ± 49.06	604.13 ± 29.8
DM, phase, °	− 12.92 ± 0.84	− 15.15 ± 0.94	− 18.01 ± 1.01	− 21.33 ± 1.3	− 25.56 ± 1.9	− 44.47 ± 2.93	− 57.2 ± 2.42
SC, modulus, Ω	2218.13 ± 111.28	2095.76 ± 124.04	1949.27 ± 115.22	1785.06 ± 68.81	1598.92 ± 51.92	947.54 ± 36.92	681.31 ± 31.54
SC, phase, °	− 14.23 ± 0.69	− 23.57 ± 2.01	− 33.98 ± 3.81	− 42.73 ± 3.45	− 51.3 ± 6.62	− 67.56 ± 1.51	− 75.12 ± 1.39
NR, modulus, Ω	931.24 ± 29.83	892.47 ± 31.69	872.75 ± 28.52	855.47 ± 34.42	814.67 ± 24.62	766.68 ± 30.79	645.17 ± 19.78
NR, phase, °	− 10.82 ± 0.82	− 12.26 ± 0.98	− 14.23 ± 0.92	− 16.86 ± 0.96	− 22.12 ± 0.97	− 41.2 ± 1.66	− 57.09 ± 1.79

*SD* standard deviation, *PLL* posterior longitudinal ligament, *DM* dura mater, *SC* spinal cord, *NR* nerve root

In general, a marked demarcation among the values of the different tissues was found, although modulus and phase did not present the same rate of variation with frequency. The behavior of the tissues in modulus and phase was different within the first group. For example, for the former, the values varied between sub-endplate cortical bone and annulus fibrosus, while, in the latter, variations were seen between sub-endplate cortical bone and PLL. Nevertheless, within the second group, for both modulus and phase, the bioelectrical impedance values varied between the spinal cord and nerve root. Additionally, the same changing trend that the modulus and phase values decreased with increasing frequency was repeated in every tissue.

**Fig. 5:**
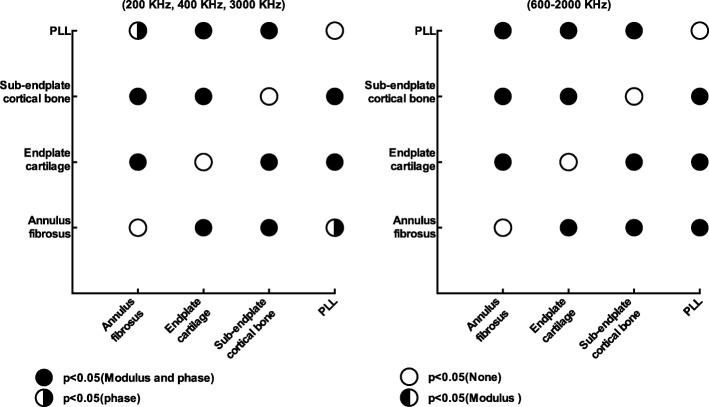
Comparison within tissues (annulus fibrosus, endplate cartilage, sub-endplate cortical bone, and posterior longitudinal ligament) over the whole frequency range. The statistical significance is defined by critical values of the post hoc test

**Fig. 6:**
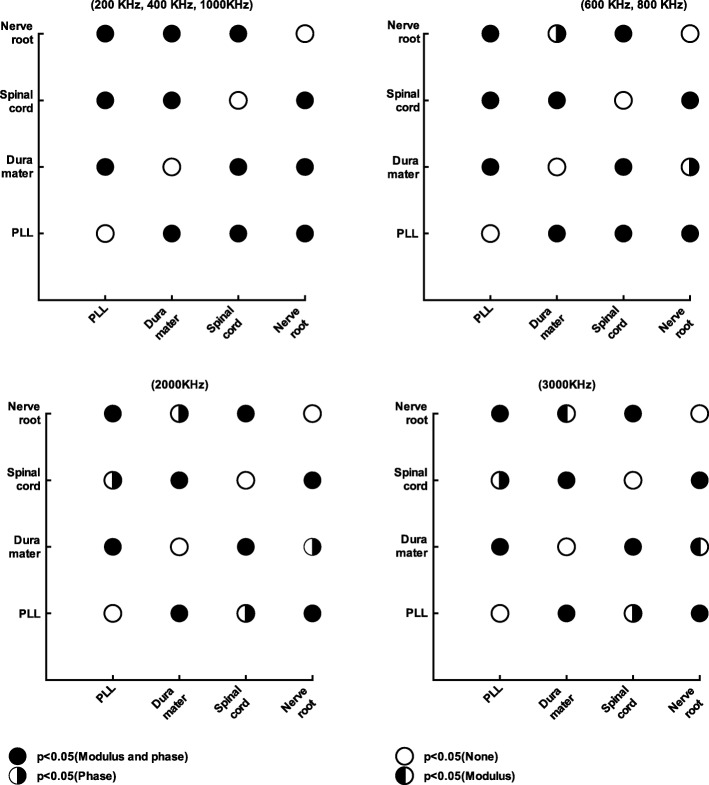
Comparison within tissues (posterior longitudinal ligament, dura mater, spinal cord, and nerve root) over the whole frequency range. The statistical significance is defined by critical values of the post hoc test

Differences in bioelectrical impedance were statistically significant in 42 tissue pairs (*p* < 0.05 for either modulus or phase or both) within the former group (Fig. 5) and 42 (*p* < 0.05 for either modulus or phase or both) within the latter group (Fig. 6). For the first group, 42 comparisons were statistically significant in phase, 39 in modulus, and 39 in both. There were no cases where a tissue could not be discriminated either in modulus or in phase. For the second group, 37 comparisons were statistically significant in modulus, 41 in phase, and 36 in both. In general, within each group, over the frequency range of 200–3000 kHz, the two tissues can be distinguished using either the modulus, phase, or both.

## Discussion

To the best of our knowledge, this is the first study to explore if bioelectrical impedance (modulus and phase) would be helpful in discriminating PLL and dura tissues in ACDF surgery. Our data suggest that, within each group, it is always possible to discriminate one tissue with respect to another at a certain frequency based on modulus, phase, or both.

More and more authors now recommended that degenerative or hypertrophic PLL should also be removed after resection of the herniated disc [7]. A recent clinical study conducted by Bai et al. described the benefit of removal of degenerative PLL in ACDF procedures for CSM [17]. Wang et al. also reported that more decompression of the spinal cord was obtained after conventional removal of the PLL. According to the MR study, the diameter of the spinal cord in PLL removed group was greater than that of the PLL preserved group [7]. However, the exposure during PLL removal process was restricted and was associated with high risk of iatrogenic trauma to surrounding tissues, including the spinal cord, nerve roots, dura, and dura mater [18, 19]. These intraoperative adverse events are partly caused by the inability to discriminate PLL, dura mater, spinal cord, and nerve root. In the present study, the results suggested that, among PLL, dura mater, spinal cord, and nerve root, for every comparison, a statistically significant difference was reported in modulus, phase, or both at every frequency point. The bioelectrical impedance signal obtained during PLL resection procedure could help the surgeons judge the surgical state and enhance safe decompression.

Cage subsidence has been frequently reported after ACDF surgery, which could lead to loss of foraminal height, graft extrusion, kyphotic deformity, pseudarthrosis, and recurring nerve root compression [20, 21]. In a recent systematic review, Noordhoek et al. reported that the overall incidence of subsidence was about 21% among patients undergoing ACDF using a cage [20]. According to Lim’s biomechanical study, the mechanical strength of the graft-endplate interface was significantly relevant to the integrity of the endplate condition, and sub-endplate cortical bone must be preserved from penetration to avoid graft subsidence [21]. Therefore, it is crucial to discriminate tissues clearly during disc removal process. In this study, the data suggested that a statistically significant difference could be found between endplate cartilage and sub-endplate cortical bone in both modulus and phase, at every frequency point. Therefore, the real-time feedback system based on bioelectrical impedance could provide useful information for surgeons while resecting the disc.

Accordingly, robot-assisted minimally invasive surgery (RMIS) has gained momentum in spine surgery as spine surgeons attempt to harness the potential benefits of RMIS. RMIS techniques for the management of spinal disorders are beneficial in preserving muscle mass, reducing soft-tissue dissection, decreasing intraoperative blood loss, and decreasing the physiological stress associated with surgery and duration of hospital stay [22]. RMIS may hold great promise for improving the accuracy and dexterity of a surgeon, but it has some critical limitations which include but are not limited to the complex anatomy and proximity to important neurovascular structures (especially in the cervical spine), registration error, and overly burdened visual channels [23]. During RMIS, surgeons mostly operate relying on their experience and the visual display with no other feedback. The notion of coupling multiple feedback systems and surgical robotics is intuitive for allowing clear tissue discrimination and accurate manipulation, especially when the visual feedback is deteriorated in RMIS, for example when the camera’s view is clouded by fluids or by the smoke generated from the electrosurgical hook operations [23]. In these circumstances, despite the advanced technologies mentioned above, there is a need for an auxiliary sensory channel in addition to the visual channels that will enhance safe PLL resection during robot-assisted minimally invasive ACDF surgery. The advantages of bioelectrical impedance measurement and spindly knife-type structure make it suitable to be integrated with the surgical robot or minimally invasive devices and work in a limited surgical field during PLL resection. The bioelectrical impedance information acquired from the sensor can be an auxiliary channel for the surgeons to discriminate tissue types without any previous knowledge of the tissue properties, which is valuable not only in traditional open surgery but also in RMIS.

Several limitations of this study should be mentioned. Although the contact force of electrodes had been kept as constant and gentle as possible, an appropriate range of force was needed to prevent the tissue from damage. In the future, this problem can be solved by integrating with the specially designed mechanism that can produce a constant output force. Additionally, tissue discrimination based on bioelectrical impedance had been verified within the frequency range of 200–3000 kHz; however, whether it is feasible along other frequency range needs further investigation. Finally, experiments on other animal species, cadavers, or in a clinical scenario are warranted.

## Conclusions

At certain frequency points, the modulus and phase of tissues relevant to disc removal and PLL resection in ACDF surgery are significantly different. The system used in this study has the potential to provide additional feedback via biomedical impedance to facilitate safe decompression in ACDF surgery, especially in RMIS.

## Data Availability

The datasets generated and analyzed during the current study are available from the corresponding author on reasonable request.

## Abbreviations

ACDF: Anterior cervical discectomy and fusion
CSM: Cervical spondylotic myelopathy
LCR: Inductance-capacitance-resistance
PLL: Posterior longitudinal ligament
RMIS: Robot-assisted minimally invasive surgery
SD: Standard deviation
